# Commentary: Evaluation of post-COVID mortality risk in cases classified as severe acute respiratory syndrome in Brazil: a longitudinal study for medium and long term

**DOI:** 10.3389/fmed.2025.1557589

**Published:** 2025-06-20

**Authors:** Julio Croda

**Affiliations:** ^1^Faculdade de Medicina, Universidade Federal de Mato Grosso do Sul, Campo Grande, MS, Brazil; ^2^Department of Epidemiology of Microbial Diseases, Yale School of Public Health, New Haven, CT, United States; ^3^Fundação Oswaldo Cruz, Campo Grande, MS, Brazil

**Keywords:** vaccination, COVID-19, Brazil, mortality, confounders, biases, hospitalization

## Introduction

The study by Rodrigues and Andrade (1) aimed to investigate factors associated with post-COVID mortality among cases of severe acute respiratory syndrome (SARS) in Brazil from 2020 to 2023. Using retrospective cohort data from SIVEP-GRIPE, they applied multiple survival analysis models (Cox proportional hazards, mixed-effects Cox, and frailty Cox) to assess medium and long-term mortality risks.

Key findings included that COVID-19 vaccination reduced mortality by 8% in the medium term, but paradoxically, vaccination was associated with an almost two-fold increase in long-term mortality risk among those vaccinated with one or two doses. The study concluded that while vaccines offered protection within the first year after infection, this effect reversed thereafter.

Given the relevance of such findings for public health policy and vaccine confidence, it is essential to critically assess the methodological robustness of this study. This commentary identifies several key methodological flaws that limit the validity of the conclusions.

## Subsections relevant for the subject

### Database limitations

The study exclusively relied on the SIVEP-GRIPE database, which is primarily designed for surveillance of acute severe respiratory infections, not for longitudinal mortality tracking. This raises concerns regarding data completeness and accuracy, particularly in capturing deaths that occur after hospital discharge.

Moreover, common data entry errors in SIVEP-GRIPE, such as incorrect recording of dates, may explain the abrupt changes observed in mortality trends over time. For instance, misentries involving the year could erroneously classify early deaths as long-term events.

Integration with Brazil's Mortality Information System (SIM) through record linkage would enhance the validity of mortality data by providing a more complete and precise assessment of outcomes. The SIM database systematically captures death certificates and is recognized as the gold standard for mortality surveillance in Brazil (2).

### Limited sample selection and biases

The study exclusively used data from the SIVEP-GRIPE database, selecting only individuals with a minimum interval of 3 months between COVID-19 symptom onset and death. This approach reduced the sample size to ~5,000 cases out of over 700,000 recorded deaths, introducing a significant selection bias and limiting the generalizability of the findings (1).

Additionally, survival bias, or immortal time bias, presents a significant challenge. High-risk individuals often die earlier, leaving healthier individuals for long-term analysis. This imbalance can result in the underestimation of mortality risks among unvaccinated individuals who survive longer, complicating the interpretation of outcomes (3).

### Inadequate control of confounders

Although the study employed Cox regression models, it failed to adequately account for critical confounders, including disparities in healthcare access, specific comorbidities, and socioeconomic factors. These unaddressed confounders likely influenced the observed outcomes, undermining the study's validity (4).

Furthermore, the study's retrospective cohort design, which focused on hospitalized patients, is suboptimal for evaluating vaccine effectiveness. Population-based cohort studies or test-negative case-control designs are more robust alternatives for assessing vaccine effectiveness (5).

### Speculative conclusions

The study hypothesized an increased long-term mortality risk among vaccinated individuals, attributing it to potential adverse events of vaccines or immune system impacts. However, these claims lack robust evidence and do not consider alternative explanations, such as preexisting comorbidities or unequal access to healthcare services (1). Additionally, the absence of detailed information on vaccine types and timing relative to hospitalization further weakens the analysis of causal relationships (2).

## Discussion

The study raises an important question about long-term COVID-19 mortality risks but is constrained by significant methodological flaws. Selection and survival biases, database limitations, and inadequate confounder control reduce the reliability of its findings. The conclusions, particularly regarding vaccine-related risks, should therefore be interpreted with caution.

Future research should address these limitations by integrating comprehensive databases like SIM, rigorously validating statistical models, and employing robust study designs such as test-negative case-control studies (6). These approaches are essential for generating reliable evidence to inform public health policies.

## Conclusion

This study highlights the need for further exploration of long-term mortality risks associated with COVID-19. However, addressing its methodological limitations will be crucial for advancing our understanding and improving the reliability of future research on vaccine safety and effectiveness.
